# Ciliary body thickness changes after preoperative anti-inflammatory treatment in rhegmatogenous retinal detachment complicated by choroidal detachment

**DOI:** 10.1007/s00417-017-3673-2

**Published:** 2017-05-10

**Authors:** Yassine Alibet, Galyna Levytska, Nicolay Umanets, Natalya Pasyechnikova, Paul B. Henrich

**Affiliations:** 1 The Filatov Institute of Eye Diseases and Tissue Therapy AMS of Ukraine, Frantsuzkyi Boulevard 49/51, Odessa, 65061 Ukraine; 2 Department of Ophthalmology, University of Basel, P.O. Box, CH-4012 Basel, Switzerland; 3 Department of Ophthalmology, Winterthur Cantonal Hospital, Winterthur, Switzerland; 4 Centro Avanti, Lugano, Switzerland

**Keywords:** Rhegmatogenous retinal detachment, Retinal detachment, Choroidal detachment, Ciliary body edema, Triamcinolone acetonide, Perfluoropropane

## Abstract

**Background:**

The literature is scant on the state of the ciliary body, its role in the development of rhegmatogenous retinal detachment (RRD) complicated by choroidal detachment (CD), and on ciliary body changes following the treatment aimed at resolving concomitant inflammation and choroidal attachment. This study assesses the anatomical position and thickness of the ciliary body and investigates the ciliary body changes after anti-inflammatory pre-vitrectomy treatment in RRD complicated by CD.

**Methods:**

Forty-nine patients (49 eyes) with RRD complicated by CD underwent standard ophthalmological examination (including visual acuity assessment, biomicroscopy, ophthalmoscopy, and ocular tonometry) and ultrasound biomicroscopy of the ciliary body, choroid, and retina both before and following anti-inflammatory pre-vitrectomy treatment.

**Results:**

At baseline, all subject eyes had ciliary body edema and detachment extending into the choroid. Ultrasonographic ciliary features included ciliary body edema and disorganization of the supraciliary layer of the pars plana, which was evident by the presence of multiple small oblique fibers. In all subject eyes, the treatment resulted in reattachment of the choroid and the ciliary body as well as a reduction in ciliary body edema (total mean ciliary thickness reduced from 0.83 (0.09) to 0.65 (0.09) mm, with a difference of 0.18 (0.07) mm, *P* < 0.001).

**Conclusions:**

Preoperative anti-inflammatory treatment in RRD complicated by CD results in restoration of the anatomical position of the ciliary body and a statistically significant reduction in ciliary body edema.

## Introduction

The prognosis for rhegmatogenous retinal detachment (RRD) complicated by choroidal detachment (CD), marked hypotony and intraocular inflammation is rather unfavorable, which is confirmed by poor anatomical and functional outcomes of the treatment and by high redetachment rates [1, 2]. Therefore, patients with this form of retinal detachment are usually excluded in multicenter prospective studies of the efficacy of treatment for RRD [3].

Primary vitrectomy has been recommended for the management of choroidal detachment associated with retinal detachment [4]. It has been reported that administration of oral steroids (prednisolone, 1 mg per kg) before primary vitrectomy in eyes with the disorder improves reattachment rates [5, 6]. Preoperative treatment of such eyes with intravitreal injections of triamcinolone acetonide (TA), either combined with expansile gases in especially severe cases [7], or alone [6, 8] has a number of advantages.

The aim of preoperative treatment of such eyes is resolution of choroidal detachment and signs of concomitant intraocular inflammation. Anti-inflammatory treatment has been shown to also result in a significant decrease in hypotony [5–10], thus evidencing the restoration of the function of the ciliary body.

The literature is, however, scant on morphological ciliary features in combined RRD and CD [11, 12], and, to the best of our knowledge, the changes in the ciliary body following anti-inflammatory treatment have not been investigated.

The aim of the study was to assess the anatomical position and thickness of the ciliary body and to investigate the ciliary body changes after anti-inflammatory pre-vitrectomy treatment in RRD complicated by CD, intraocular inflammation, and hypotony.

## Methods

This prospective non-randomized interventional clinical trial involved 49 patients (22 men and 27 women; 49 eyes; age, 24 to 83 years) with RRD complicated by concomitant CD and intraocular inflammation.

Study eyes were treated as per the methodology protocol approved by the ethical committee of the Filatov Institute in 2012 and by the National Academy of Medical Science of Ukraine 2 years later (Information Bulletin No. 37 of the year 2014, p.145, based on Pat. of Ukraine №81,704 issued 10.07.2013. Method for treatment of rhegmatogenous retinal detachment complicated by choroidal detachment. Authors: Levytska G, Putiienko O, Abdulkhadi M. Owner: State Institution Filatov Institute of Eye Diseases and Tissue Therapy NAMS of Ukraine).

Exclusion criteria were history of previous ocular inflammatory diseases, ocular trauma or retinal surgery. All patients underwent standard ophthalmological examination including visual acuity assessment, biomicroscopy, ophthalmoscopy, and ocular tonometry. Additionally, ultrasound biomicroscopy (UBM) of the ciliary body, choroid and retina was performed.

**Fig. 1:**
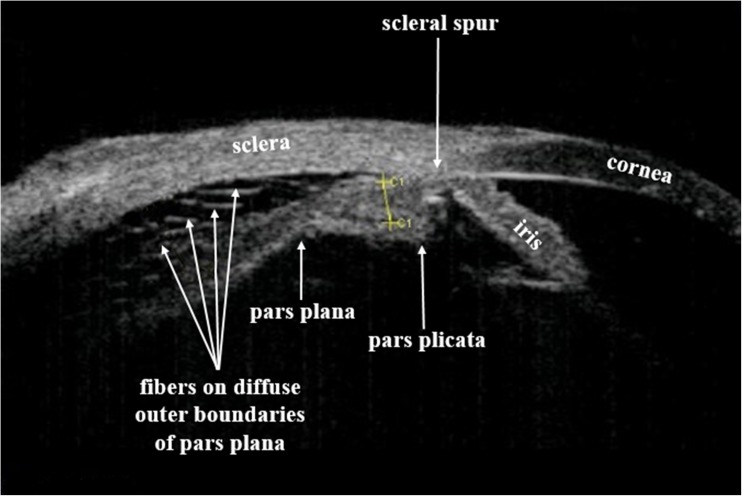
Ultrasound biomicroscopic measurements. Edematous ciliary body is seen (ciliary body thickness (C1) measured between the ciliary processes located most closely to the scleral spur is 0.79 mm*)* with poorly defined anterior border of pars plana ciliaris and multiple small oblique fibers. The pars plicata is adherent to the sclera while the pars plana is detached along with the detached choroid

**Fig. 2:**
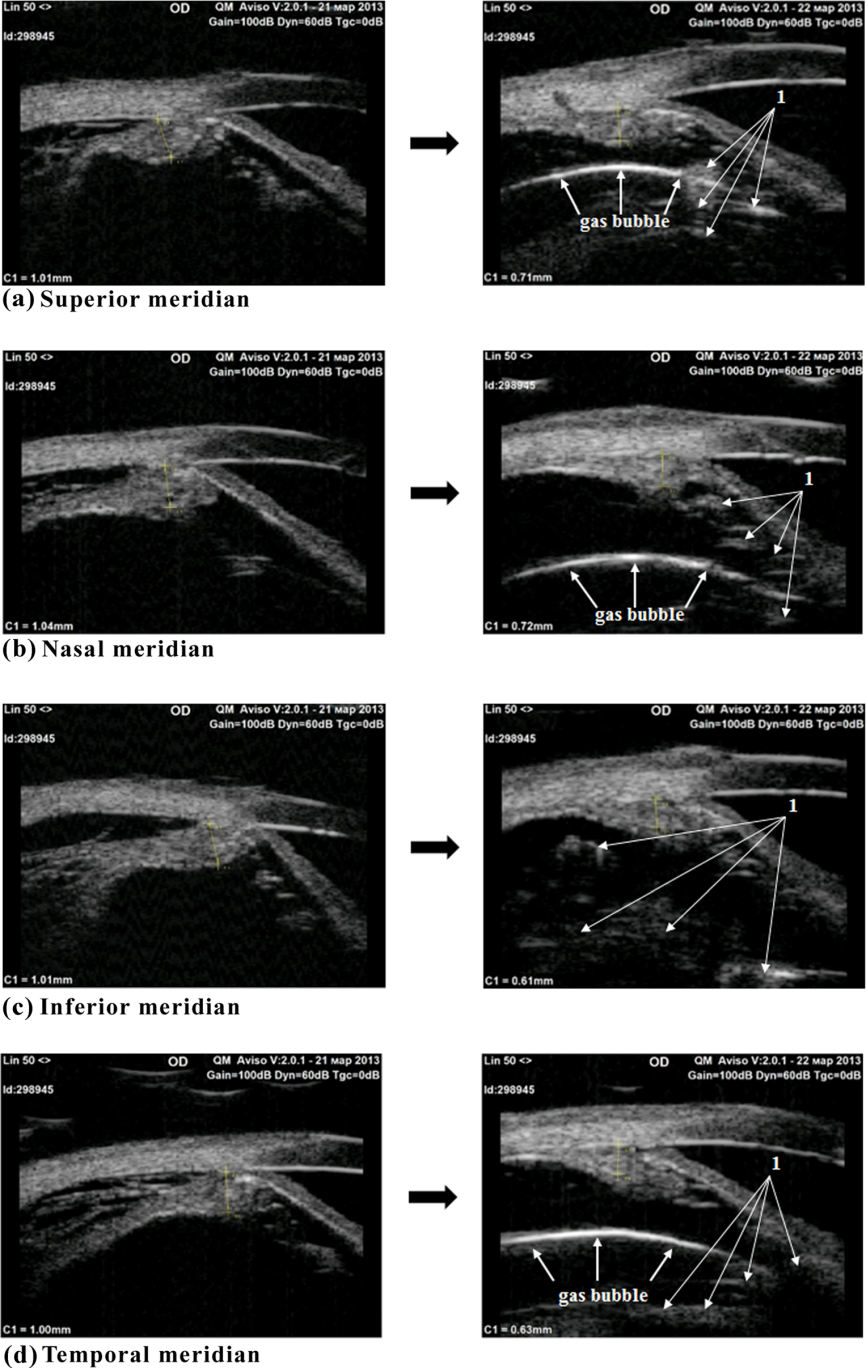
Ultrasound biomicroscopy images showing the ciliary body before (left side) and 1 day after (right side) treatment with intravitreal injection of 4 mg of triamcinolone acetonide in combination with perfluorpropane. Pretreatment (left side) images at the superior (a), nasal (b), inferior (c), and temporal (d) meridians show edematous and detached ciliary body with multiple small oblique fibers on diffuse outer boundaries; the detachment extends into the choroid. Post-treatment (right side) images at the same meridians show reduced ciliary body edema and complete attachment of the ciliary body. Additionally, triamcinolone acetonide crystals and opacities are well differentiated against the crystal background in the vitreous cavity (arrow 1). The gas bubble is localized in the scans taken in each of the meridians (except the scan taken in the inferior meridian)

Ciliary body thickness (CBT) measurements were performed under cycloplegia with phenylephrine hydrochloride 10% and cyclopentolate hydrochloride 1%, at the four cardinal meridians (i.e., the superior, inferior, nasal, and temporal) of the eye. Determination of CBT at the pars plana portion in patients with this disease is complicated due to the absence of well-defined boundaries, presence of multiple small oblique fibers on poorly defined boundaries (Fig. 1) and difficulties in determination of the projection of detached portion of the ciliary body onto the sclera. Pars plana thickness was also measured at the four cardinal meridians (i.e., the superior, inferior, nasal, and temporal) of the eye (Fig. 2).

**Fig. 3:**
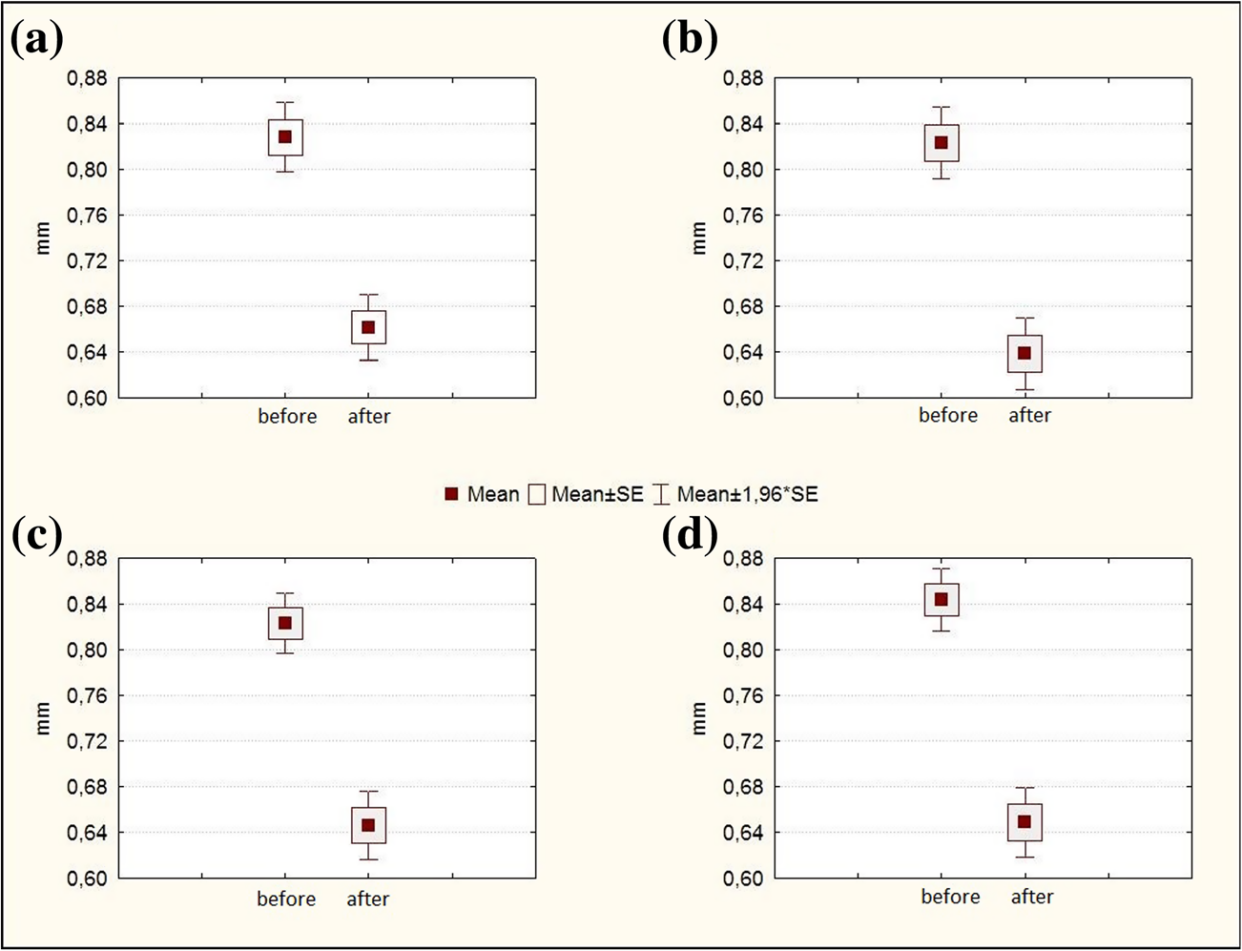
Ciliary body thicknesses in the superior (a), nasal (b), inferior (c), and temporal (d), meridians in rhegmatogenous retinal detachment complicated by choroidal detachment before and after preoperative anti-inflammatory treatment. The ordinate displays the ciliary body thicknesses in mm. The upper and lower margins of the boxes in this standard box-and-whisker diagram represent the 25th and the 75th, the central line inside the box the 50th percentile (median). The whiskers mark the minimum and the maximum

We recommend measuring CBT in between the ciliary processes located most closely to the scleral spur (Pat. of Ukraine №105,205, 10.03.2016. Method for determining the ciliary body thickness at its detachment. Authors: Levytska G., Kovalchuk A., Alibet Y. Owner: State Institution Filatov Institute of Eye Diseases and Tissue Therapy NAMS of Ukraine), since it is at the ciliary process portion of the ciliary body that ciliary body boundaries are best defined (Fig. 1). No significant difference was revealed between the mean CBT values at different locations, *P* = 0.35. Mean CBT in the four meridians in RRD complicated by choroidal detachment before and after preoperative treatment are presented in Fig. 3. The difference in mean CBT varied from 0.17 mm to 0.19 mm, with the SD varying from 0.09 to 0.1 (Fig. 3).

Given no statistically significant difference in the CBT at the four cardinal meridians, the changes in the thickness values over time were assessed based on the total mean CBT values.

We used the Quantel Medical Aviso UBM unit (Quantel Medical, Clermont-Ferrand, France) with a 50-MHz linear probe (axial resolution: 35 μm; lateral resolution: 60 μm). Patients had ultrasound biomicroscopy performed while positioned supine with head-of-bed elevation, both before and 1 to 4 days after intravitreal injection.

Prior to vitrectomy, under the above-mentioned cycloplegic conditions and following preoperative topical treatment with 0.1% dexamethasone and cyclopentolate hydrochloride 1% eye drops, study eyes received anti-inflammatory therapy, with 4 mg of (0.1 mL) TA intravitreally injected either alone (30 eyes) or in combination with 0.4 to 0.8 mL of perfluorpropane until IOP became normotensive (19 eyes). This treatment aimed at preoperative resolution of choroidal detachment and intraocular inflammation to decrease the risk of intra- and postoperative complications in retinal detachment surgery.

Statistical analyses were conducted using Statistica 10.0 (StatSoft, Tulsa, OK, USA) software. The parametric Student *t* test was used for unpaired samples. The level of significance *p* ≤ 0.05 was assumed. Data are presented as mean (with standard deviation (SD) in parentheses).

## Results

Baseline UBM revealed ciliary body edema with ciliary body and choroidal detachments in all patients.

It is noteworthy that in this category of patients, ciliary body detachment was characterized by the detachment of the pars plana only, whereas attachment of the pars plicata to the sclera was maintained. Therefore, no connection was observed between the subchoroidal spaces and the anterior chamber, which is a feature that distinguishes ciliary body detachment from other ocular pathologies with RRD (Fig. 1).

Choroidal detachment in all subject eyes was characteristic in that it extended to the equator or slightly posterior to the equator, without involvement of the posterior pole. The maximum height of the choroidal detachment was observed in cases with the maximum extent of this detachment.

The mean CBT at the above-mentioned four meridians varied from 0.82 mm to 0.84 mm, with the SD varying from 0.08 to 0.1. Additionally, no significant difference was revealed between the mean CBT values at different locations,

*P* = 0.34. Therefore, it is possible to assess changes in the CBT based on the total mean value of this index at different meridians for all subject eyes, which was 0.83 (0.09) (range, 0.68 to 1.05 mm; median, 0.82 mm) in the study reported here.

The course of retinal detachment in eyes of the study was remarkable for marked hypotony, with a mean intraocular pressure (IOP) level of 6.9 (1.5) mm Hg (range, 5 to 11 mmHg). Choroidal detachment in three or more quadrants was found in 63.3% of cases, with a mean height of the detachment of 3.86 (2.13) mm (range, 0.3 mm to 8.5 mm).

As early as 1 to 2 days following an intravitreal injection, no signs of intraocular inflammation (ciliary tenderness, conjunctival injection, and posterior synechiae) were found in any treated eye. One to 4 days following an intravitreal injection, the IOP increased from baseline of 6.9 (1.5) Hg to 13.3 (0.9) mm Hg (*P* = 0.0001).

Postoperative ultrasound biomicroscopy revealed a reduction in the signs of intraocular inflammation and an improvement in IOP levels. Ciliary body and choroidal reattachment, as well as a reduction in ciliary body edema was achieved in all 49 subject eyes. In all subject eyes, UBM revealed a reduction in the ciliary body edema following intravitreal injection of TA alone or in combination with perfluorpropane. After treatment, the total mean CBT was 0.65 (0.09) mm (range, 0.51 mm to 0.93 mm; median, 0.63 mm).

The total mean ciliary body thickness value thus changed significantly from baseline following treatment, showing a significant reduction in ciliary body edema [baseline 0.83 (0.09) mm vs. post-treatment 0.65 (0.09) mm, with a difference of 0.18 (0.07) mm; *P* < 0.0001].

## Discussion

Rhegmatogenous retinal detachment (RRD) complicated by choroidal detachment (CD), marked hypotony and intraocular inflammation is associated with comparatively poor anatomical and functional outcomes following vitreoretinal interventions, including high redetachment rates [1, 2]. Despite a rather scant body of evidence, the literature suggests that administration of oral steroids (prednisolone, 1 mg per kg) before primary vitrectomy may improve reattachment rates [5, 6] in this situation. Because of reduced systemic side effects, intravitreal application TA has also been advocated, either alone [6, 8] or combined with expansile gases in especially severe cases [7].

The intravitreal anti-inflammatory treatment used in this study aimed to resolve choroidal detachment by means of improving the competence of the blood-retinal barrier. Anti-inflammatory treatment has been shown also to result in a significant improvement of hypotony [5–10], thus evidencing the restoration of the function of the ciliary body. In severe cases, intravitreal C3F8 gas was added to further counteract ocular hypotony.

Previously, preoperative intravitreal TA demonstrated positive outcomes in eyes with RRD combined with CD [1, 2], where “the uveitis of all 28 eyes” improved within a day [2] and CD “disappeared in most of cases within 10 days TA injection” [1]. In addition, it is known that the use of TA allows the ophthalmologist (1) to reduce systemic side effects of steroids and (2) to reduce the period of preoperative (i.e., pre-vitrectomy) preparation.

Additional intravitreal gas injections are intended to further normalize ocular hypotony. Although the use of intravitreal air could have been an alternative to C3F8, treatment was performed as per the methodology protocol that was approved in 2012 by the ethical committee of the Filatov Institute and later on by the National Academy of Medical Science of Ukraine (Information Bulletin No.37 of the year 2014, p.145, based on Pat. of Ukraine №81,704 issued 10.07.2013.).

Resolution of intraocular inflammation and choroidal detachment, as well as normalization of the IOP were the basic criteria for the efficacy of preoperative treatment.As early as 1 to 2 days following an intravitreal injection, no signs of intraocular inflammation (ciliary tenderness, conjunctival injection, and posterior synechiae) were found in any treated eye. One to four days following an intravitreal injection, the IOP increased from baseline of 6.9 (1.5) Hg to 13.3 (0.9) mm Hg (*P* = 0.0001), indirectly evidencing an improvement in the function of the ciliary body (and particularly restoration of intraocular fluid production).

Visual acuity improvement, a conventional criterion for treatment efficacy, was not applicable in our study, as preoperative treatment was not intended to improve vision directly, but to create improved conditions for uncomplicated vitrectomy.

It is noteworthy that reattachment of the ciliary body and choroid was achieved at each meridian (i.e., in the inferior eye also rather than only at the superior meridian, in the area lying close to gas bubble pressure) with the patient’s head positioned vertically. This finding confirms that our treatment approach of this specific form of RRD is justified pathogenetically.

The therapeutic effect obtained in the study advocates one of the theories of the pathogenesis of the development of choroidal detachment. The mechanism of the development of choroidal detachment in ocular trauma and in a complicated glaucoma surgery has been described in detail in [13, 14] and [15], respectively.

However, the development of choroidal detachment in RRD follows another mechanism, which has been significantly less studied. Jarret reported a hypothesis regarding the development of choroidal detachment in RRD more completely than others; his paper [12] involved the analysis of 47 relevant cases found during a 12-year observation period. RRD is known to be accompanied by a blood ocular barrier breakdown [16, 17]; it is the latter that is a casual event of increased ocular vascular permeability, transudation or exudation of fluid into the extracellular spaces, accumulation of fluid in the suprachoroidal spaces, and ciliary body and choroidal detachment. This in turn results in decreased aqueous production and development of acute hypotony, thus completing the vicious cycle [12]. An increased absorbing surface of the retinal pigment epithelium exposed to the subretinal fluid is another cause for the presence of marked hypotony in RRD [18]. This might explain the fact that in our study, the IOP level was restored incompletely, just to 13.1 (0.8) mm Hg despite successful re-attachment of the ciliary body and choroid.

Seelenfreund et al. [19] believe that the height and extent of choroidal detachment might depend on the degree of vitreous contraction and state of choroidal vessels. We find this hypothesis likely; it explains why CD is more often found in elder RRD patients than in those of other age groups. We failed to find any literature on the morphological ciliary features in ciliary body detachment in eyes with RRD. It has been reported that following scleral buckling surgery for RRD, ciliary body changes [20] involved ciliary edema, which was in some cases accompanied by increased IOP levels [21] due to alterations in the anterior chamber angle within 3 days after surgery [21, 22]. These alterations are caused not only by postoperative edema, but also by the presence of sclera buckling material which displaces the ciliary body anteriorly. In the following days, as inflammation subsided, approximately by day 28, the initial ciliary body thickness and ocular hydrodynamic indices restored [20].

To what extent the improvement of the ciliary body edema was caused by the anti-inflammatory effect of treatment or the increase of intra-ocular pressure cannot be deducted from our results. An anti-inflammatory effect would be more consistent with Jarret’s hypothesis, while previous studies of this study group suggest there may be a combined effect. (Information Bulletin No.37 of the year 2014, p.145, based on Pat. of Ukraine №81,704 issued 10.07.2013. Method for treatment of rhegmatogenous retinal detachment complicated by choroidal detachment. Authors: Levytska G, Putiienko O, Abdulkhadi M. Owner: State Institution Filatov Institute of Eye Diseases and Tissue Therapy NAMS of Ukraine).

The results of this study are limited by its non-randomized nature. We believe, however, that evidence from this trial is conclusive, raising the question wether randomized trials with an untreated control group would be ethically acceptable, given the superiority of preoperative anti-inflammatory treatment in the present trial.

## Conclusion

Preoperative anti-inflammatory treatment in RRD complicated by CD resulted in restoration of the anatomical position of the ciliary body and a statistically significant reduction in ciliary body edema. The mechanism of the development of choroidal detachment in the presence of RRD is likely to be based on a blood-ocular barrier breakdown, which explains the high efficacy of the treatment used.
